# A case of thoracic pneumatosis due to severe coughs and tracheal tube displacement induced by tracheal tube size mismatch

**DOI:** 10.1186/s40981-019-0227-0

**Published:** 2019-02-09

**Authors:** Yuka Tachiiri, Satoki Inoue, Masahiko Kawaguchi

**Affiliations:** Department of Anesthesiology and Division of Intensive Care, Nara Medical University, 840 Shijo-cho Kashihara, Nara, 634-8522 Japan

**Keywords:** Pneumothorax, Intubation, Size mismatch

## Abstract

**Background:**

Thoracic pneumatosis during mechanical ventilation may be life-threatening. We encountered a patient with thoracic pneumatosis after frequent displacement of the tracheal tube with an overinflated cuff.

**Case presentation:**

We admitted a 62-year-old man to the intensive care unit (ICU) due to respiratory failure. We secured his airway using a cuffed 8.5-mm tracheal tube. However, air leakage did not stop with the regular intracuff pressure (25 cm H_2_O) because the diameter of his trachea was too large for the tracheal tube inserted. In addition, a chest X-ray examination revealed rostral tube displacement. Therefore, we applied a higher intracuff pressure (35 cm H_2_O) to prevent air leakage and tracheal tube movement. However, severe coughing episodes developed, and 3 days after ICU admission, a chest X-ray and CT scan revealed pneumomediastinum and pneumothorax. We did not have larger tracheal tubes in stock. We decided to use a tracheostomy tube instead, which we expected to be placed securely and to prevent tube displacement. After tracheostomy, the severe coughing episodes became infrequent. Finally, we weaned the patient from mechanical ventilation 12 days after ICU admission.

**Conclusions:**

The clinical signs and symptoms in our patient point to tracheal tube size mismatch as the cause of pneumothorax.

## Background

Overinflation of the tracheal tube cuff and a sudden movement of the tube are common causes of tracheal rupture [1]. Tracheal rupture causes pneumomediastinum, pneumothorax, and subcutaneous emphysema, which may all be life-threatening during mechanical ventilation. In addition, high ventilation pressures and global or regional overdistension of the airways are also responsible for most cases of barotrauma [2]. We encountered a patient developing pneumothorax and pneumomediastinum after cough-induced barotrauma due to irritative tube movement, probably due to the tracheal tube size mismatch in spite of an overinflated cuff.

## Case presentation

We obtained the written consent of the patient’s next of kin; however, the institutional review board approval was exempted because our case report did not pose any ethical problems and we did not identify the patient. A 62-year-old man (height, 165 cm and weight, 43 kg) with chronic heart failure was admitted to the intensive care unit (ICU) because of respiratory failure. Immediately before admission, we stabilized his airway using a cuffed 8.5 mm tracheal tube (TaperGurd Evac™, Covidien, Tokyo, Japan). His chest X-ray showed bilateral infiltrates, and his blood gas showed evidence of a moderate level of acute respiratory distress syndrome. Immediately after beginning mechanical ventilation, we applied high (15 cm H_2_O) positive end-expiratory pressure (PEEP) combined with low tidal volume. We used positive pressure ventilation to achieve around 300 mL of tidal volume, which required 11–13 cm H_2_O of driving pressure. However, the air leakage did not stop with the regular intracuff pressure (25 cm H_2_O) due to the tracheal tube size mismatch (the diameter of his trachea was too large for the tracheal tube used). The patient’s trachea had an anteroposterior diameter of 28.6 mm. In addition, we observed rostral tube displacement on chest X-ray examinations. Therefore, we applied a higher intracuff pressure (35 cm H_2_O) to prevent air leakage and tracheal tube movement. We also applied moderate sedation (Richmond Agitation-Sedation Scale [RASS] − 2 to − 3) until the patient’s condition was stabilized, and he would only rarely cough spontaneously.

**Fig. 1:**
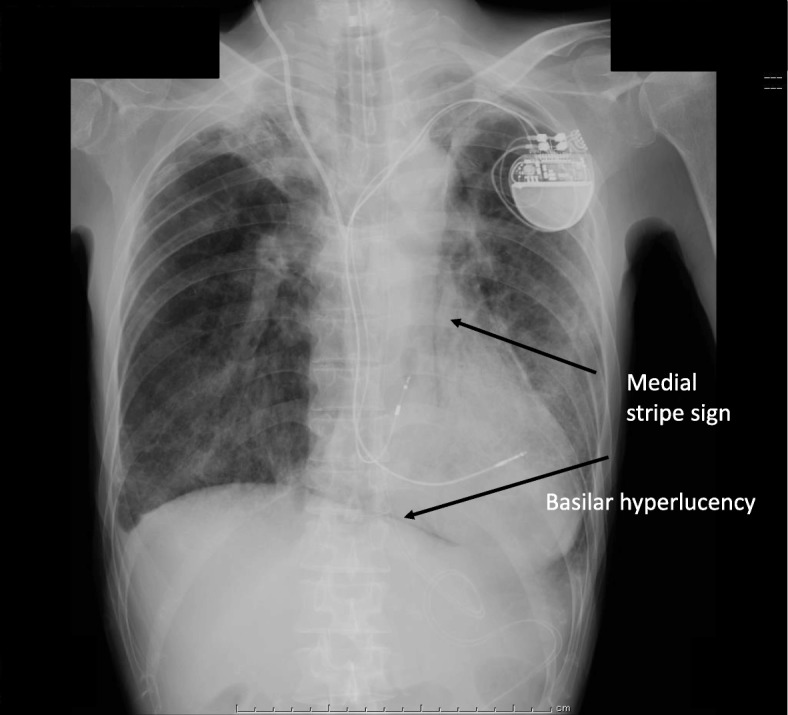
Pneumatosis. Chest X-ray shows a radiolucent outline around the heart and mediastinum. The arrows show a medial stripe sign and basilar hyperlucency

**Fig. 2:**
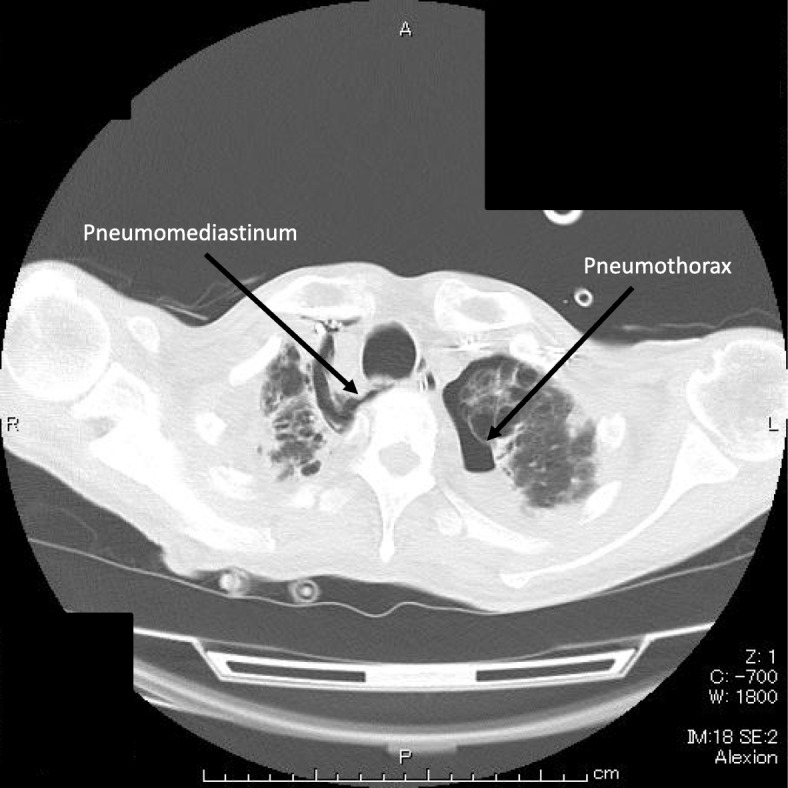
Thoracic pneumatosis. The arrows show pneumomediastinum and pneumothorax

The patient’s respiratory status improved gradually. We gradually reduced the PEEP to 8 cm H_2_O over the next 2 days. Then, we switched his sedation level from moderate to light sedation (RASS 0 to − 1). However, severe coughing episodes occurred frequently accompanied by oxygen desaturations that required transient deeper sedation each time. Finally, we decided to reintroduce moderate sedation, but we still observed occasional desaturations with severe coughing episodes. A chest X-ray, 3 days after ICU admission, showed a medial stripe sign and basilar hyperlucency indicating pneumomediastinum and pneumothorax (Fig. 1). A subsequent chest CT confirmed these thoracic pneumatosis findings (Fig. 2). We did not have larger tracheal tubes in stock at the time, and we decided to switch from the oral–tracheal tube to a tracheostomy tube, expecting it to be placed securely and to not get displaced. Four days after the ICU admission, we performed a percutaneous tracheostomy using an 8.5 mm commercially available kit (Neo Perc™, Covidien Japan, Tokyo). This time we applied a slightly lower intracuff pressure (30 cm H_2_O) to prevent air leakage. After the tracheostomy, the severe coughs seldom occurred even in the absence of sedation. The tube position was unchanged and the thoracic pneumatosis did not get worse. Thus, we monitored the patient’s condition closely without providing thoracic drainage. Finally, we weaned him from mechanical ventilation 12 days after ICU admission and discharged him from the ICU 2 days later.

## Conclusions

Our patient’s thoracic pneumatosis was probably caused by tracheal injury during positive airway ventilation because of both frequent movement of the overinflated tracheal tube cuff [1] and cough-induced barotrauma due to the irritative tube movement. The patient’s tracheal tube size mismatch may have caused the constant tube movement. The inner diameter (ID), outer diameter (OD), and the cuff resting diameter (CRD) of the tracheal tube that we used were 8.5, 12.6, and 28.6 mm, respectively. The CRD was almost the same as the patient’s tracheal diameter, a size probably insufficient to seal the trachea and allowing for tube movement. The instability of the tube may have irritated the trachea and induce coughing episodes leading to high ventilation pressures and global or regional over airway distensions [2]. Generally, an adult’s trachea has an ID of about 1.5 to 2 cm [3]. Our patient was a relatively small Japanese man, but he had a considerably large trachea. The etiology of his tracheomegaly is unknown; however, a syndrome characterized by marked tracheobronchial dilation and recurrent lower respiratory tract infections, also known as Mounier–Kuhn syndrome, has been reported [4]. The majority of patients with this syndrome present after the third decade of life, suggesting an acquired rather than a developmental anomaly [5]. Our patient had a past history of recurrent lower respiratory tract infections. Therefore, he may have suffered from this syndrome. We cannot be certain that appropriate air sealing and secured tube fixation would have been obtained with larger tracheal tube sizes. The inserted tracheostomy tube may have also been small (ID = 8.5 mm, OD = 12 mm, CRD = 30 mm), but the intratracheal movement of the tracheostomy tube was more restricted than that of the oral–tracheal tube. Thus, further deterioration did not occur.

The optimal timing for tracheostomies in mechanically ventilated patients is still debatable. Very early tracheostomy (within 4 days) may be not beneficial; however, waiting for more than 10 days may not be reasonable either [6, 7]. In our case, waiting for the resolution of the thoracic pneumatosis with oral–tracheal intubation combined with deeper sedation or neuromuscular blocking might have avoided the tracheostomy, but the extended deeper sedation of neuromuscular blocking could have induced ICU-acquired weakness, resulting in a failure of weaning from mechanical ventilation [8]. Otherwise, we might have encountered severe coughing episodes and frequent tracheal tube displacements after reintroduction of light sedation, which might have exacerbated the clinical situation. We believe that the decision to perform a very early tracheostomy played a pivotal role to improve our patient’s critical status.

In conclusion, we encountered a patient with thoracic pneumatosis due to tracheal tube size mismatch. In retrospect, an earlier tracheostomy could have prevented the development of thoracic pneumatosis.

## Abbreviations

CRD: Cuff resting diameter
ICU: Intensive care unit
ID: Inner diameter
OD: Outer diameter
PEEP: Positive end-expiratory pressure
RASS: Richmond Agitation-Sedation Scale
